# The Hospital World

**Published:** 1910-04-09

**Authors:** 


### The Hospital World.

Alderman J. S. Balfour has been re-elected chairman of the Hornsey Cottage Hospital.

Mrs. Joseph Chamberlain has been unanimously elected president of the Birmingham Lying-in Charity.

Mr. R. Shore has been re-elected chairman and the Rev. J. J. Lewis vice-chairman of the Ramsbottom Cottage Hospital.

The Earl of Lichfield has been re-elected president, and Sir Thomas Salt treasurer to the Staffordshire General Infirmary.

The Marquis of Abergavenny has been re-elected president, and Mr. R. C. Hobart has been re-elected chairman of the Tunbridge Wells Ear and Eye Hospital.

Mr. A. J. Grimwade has been re-elected hon. treasurer, and Mr. J. Alexander and Mr. F. G. Steed hon. secretaries to the St. Leonard's Hospital, Sudbury, Ireland.

Colonel Croker-King, who for six years was hon. treasurer and secretary, and has been sixteen times elected president of the Cheltenham General Hospital, was again unanimously chosen for that office at the recent annual meeting. Mr. C. L. Grundy was again elected hon. treasurer.

Lord Nunburnholme, the Rev. A. B. G. Lillingston, Mr. G. Ramsey, Mr. H. H. Sissons, Mr. E. H. Howlett, Mr. Alwyn D. Smith, and Mr. W. R. Chidley have been re-elected to the management committee of the Hull Royal Infirmary, on which Mr. J. Watson is a newly elected member.

The death of Dr. Foxwell, the senior physician of the Queen's Hospital, Birmingham, in August last, was a severe and grave loss to the hospital. It has been decided by the medical committee, with the cordial agreement of the general committee, to found a prize to his memory, and to fix a memorial tablet in the new block.

On the motion of the Chairman, seconded by Mr. Alexander Johnston, a resolution expressing the regret of the directors at the death of Sir Reginald Ogilvy was adopted at the last meeting of the Governors of the Dundee Royal Infirmary, and the clerk was instructed to send an excerpt of the minute to the relatives of the deceased baronet.

Sir Charles Whitehead (chairman), Dr. Adams, the Rev. T. W. Carr, Mr. R. Cooke, General Cumberland, Mr. R. D. de Uphaugh, Mr. T. C. Colyer Fergusson, the Rev. E. H. Hardcastle, Mr. W. Haynes, Colonel Luck, Major Lysons, Mr. H. Monckton, Mr. R. D. Turner, Mr. A. T. Waring, Mr. W. H. Whitehead, Mr. H. Morfett, the Rev. E. Best Dalison, General Sir G. Wolseley, and Mr. J. Arkcoll have been elected to the Board of Management of the Kent County Ophthalmic Hospital.

At a special meeting of the Board of Governors of the Leicester Infirmary, held on March 30, Sir Edward Wood was unanimously re-elected chairman, and Sir Arthur Hazlerigg, Bart., J.P., vice-chairman for the ensuing year. The announcement made by Mr. G. Murray Smith, who for eight years had been vice-chairman, that he could not see his way to allow himself again to be nominated by reason of his numerous engagements and his inability to be present at the meetings of the Board, was received with very great regret, and a vote of thanks expressive of his deep interest and generous support, proposed by the Chairman, seconded by Mr. Fielding Johnson, jun., and supported by Alderman Vincent, was unanimously directed to be entered on the minutes. Mr. Murray Smith announced that although he was compelled to resign the vice-chairmanship he would still be glad to serve on the Board of Governors, as well as on the House, Finance, and Drug and Medical Stores' Committees, and attend whenever possible.

The Mayor of Bolton (Mr. J. Tyas Cooper), the Rev. T. A. Chapman, Alderman W. Nicholson, J.P., Mr. W. Kevan, J.P., Mr. W. Haslam, Mr. P. C. Marsden, J.P., and Mr. F. Murton have been appointed vice-presidents of the Bolton Infirmary.

Mr. William Lintott has been asked to accept the presidency of the Horsham Cottage Hospital. It is ten years ago since he held the office. Mr. Porter, the hon. secretary, and Mr. Stott, the hon. treasurer, have been re-elected.

Messrs. Alan Broadberry, J. J. Middleton, W. R. Baddeley, J. W. Booth, G. T. Brown, A. C. Denham, H. J. Gould, W. Longhurst, J. Mitchell, the Rev. E. W. Platt, H. Rigden, G. Sweetland, H. Smith, W. T. Thompson, the Rev. H. Varley, and G. W. Wagner have been elected members of the General Council of the Lower Green Passmore-Edwards Hospital.

At the 137th anniversary meeting of the Governors of Leicester Infirmary the deep sympathy of the meeting was directed to be conveyed to Mr. S. Francis Stone, D.L., J.P. (treasurer of the institution), in the great loss he has recently sustained by the death of his third son, Mr. Lennard Chamberlin Stone.

The death of Sir Edward Wills, Bart., K.C.B., removes a good friend to the hospital world, more particularly, of course, to hospital workers at Bristol. It was he, of course, who made the Bristol Jubilee Convalescent Home possible by his gift of £25,000, overcoming every obstacle by his energy and enthusiasm. He was a hospital worker also in his capacities of first president of the Home and governor of the Bristol General Hospital.

It is proposed to build a free lying-in hospital of about 100 beds for poor women in the mill district of Bombay, for which the following gentlemen have agreed to become trustees :—Sir Jamsetji Jeejeebhoy, Bart., Sir Sassoon David, Sir Vithaldas D. Thakersey, Mr. Jamsetji Ardeshir Wadia, Mr. C. N. Wadia, Mr. Jehangir Bomanji Petit, and the Hon. Mr. Fazulbhoy Currimbhoy.

After twenty-five years' work and personal service as chairman of the committee of the Jenny Lind Infirmary, Norwich, Mr. J. J. Winter has resigned. No man ever identified himself more with his work than Mr. Winter, and there remain many of those with whom he came into contact who could tell tales of his personal kindness. One incident at least has been recorded, which we are happy to reproduce here for the benefit of Mr. Winter's many friends outside Norwich. A former hon. secretary of the infirmary (Mr. C. E. Noverre), in a letter to the local newspaper, writes : "A case of scarlet fever developing on a Sunday the matron sent to me to get an order for admission into the Fever Hospital. 'I went up to Mr. Winter's house, for then there was a great deal of red tape exercised to secure an order, especially on a Sunday. Mr. Winter . . . a strict Sabbatarian . . . listened very earnestly to my tale, and although his lunch and his family were waiting, at once said : 'You can go home quite comfortably, Mr. Noverre, I will not eat a morsel until I have seen the child safely housed at the Fever Hospital.'" This implies a great deal more than it says, and its implication is a life of personal service which, but for the lucky and generous memory of a friend, might have come to an end almost unheard of, as have, we fear, the lives of too many of the real, unhonoured workers in the hospital world. Whatever may be the reason which has led Mr. Winter to resign, it goes without saying that it is no loss of interest in the Jenny Lind Infirmary, which indeed has become a part of his life.

